# Patients’ Experience of Stigma as the Hidden Burden of Metabolic Dysfunction-Associated Steatotic Liver Disease: A Descriptive Qualitative Study with Thematic Analysis

**DOI:** 10.3390/healthcare14050579

**Published:** 2026-02-25

**Authors:** Johanne Lisa Jensen-LeBlanc, Pernille Andreassen, Mette Munk Lauridsen, Lea Ladegaard Grønkjær

**Affiliations:** 1 Department of Gastroenterology, University Hospital of Southern Denmark, 6700 Esbjerg, Denmark; johannelisa.jensen-leblanc@uwaterloo.ca (J.L.J.-L.); mette.enok.munk.lauridsen@rsyd.dk (M.M.L.); 2 The Danish National Center for Obesity, 8200 Aarhus, Denmark; pernar@rm.dk; 3 Department of Regional Health Research, University of Southern Denmark, 5230 Odense, Denmark

**Keywords:** fatty liver disease, liver diseases, metabolic syndrome, social stigma, qualitative research, patient-centered care

## Abstract

**Background/Objectives**: Metabolic dysfunction-associated steatotic liver disease (MASLD) is the most prevalent liver disease globally, closely associated with obesity and metabolic syndrome. Despite its clinical significance, patients frequently experience stigmatization from society, healthcare professionals, and family, which may exacerbate psychological distress and hinder effective care. This study aims to explore how patients living with MASLD experience and interpret different dimensions of stigma. By examining stigma across healthcare, social, and personal contexts, the study offers insights into the hidden psychosocial burden of MASLD and may inform approaches to reduce stigmatization and promote more patient-centered and equitable care. **Methods:** Between 19 November 2024 and 6 February 2025, 23 adults diagnosed with MASLD (21 women, aged 35–70+ years) completed an anonymous online questionnaire comprising 4 demographic questions and 11 open-ended questions regarding their experiences of stigma, its emotional and social impact, and strategies for managing these experiences. Responses were analyzed thematically using Braun and Clarke’s six-phase framework to identify key domains. **Results:** Three overarching themes emerged: (1) Diagnosis, Symptoms, and Medical Experiences; (2) Emotional and Social Impact; and (3) Coping Strategies and Advocacy. Most participants reported incidental diagnosis, limited or conflicting medical guidance, and feeling blamed for lifestyle choices. Several participants described anxiety and depressive symptoms, while many reported social withdrawal and self-blame, illustrating the interplay of structural, public, and self-stigma. Coping strategies included peer support, self-education, and advocacy, yet participants emphasized that lifestyle changes remained challenging without professional guidance. Many expressed a need for holistic care addressing both medical and psychosocial needs. **Conclusions:** Stigma profoundly affects patients with MASLD, influencing healthcare experiences, emotional well-being, and social interactions. Interventions should target multiple levels of stigma, incorporating education for healthcare professionals, holistic care models, and accessible support systems. Further research is needed to identify effective strategies for reducing stigmatization and improving equity of care.

## 1. Introduction

Metabolic dysfunction-associated steatotic liver disease (MASLD), formerly known as non-alcoholic fatty liver disease (NAFLD), is the most common liver disease worldwide and has become a major global health concern [1]. The global prevalence among adults has risen over the past decade, from approximately 26% to 38%, and projections suggest it may reach 55% globally by 2040 [2]. Closely associated with obesity, type 2 diabetes, and other components of metabolic syndrome, MASLD is a progressive condition that can advance from simple steatosis to cirrhosis and hepatocellular carcinoma, imposing a significant clinical burden. Although the disease remains asymptomatic in early stages, patients may experience non-specific symptoms such as abdominal discomfort, fatigue, and reduced physical functioning. As the disease progresses, complications may include liver failure, the need for transplantation, and increased mortality [3]. Beyond physical manifestations, MASLD has been associated with impaired health-related quality of life, psychological distress, and social limitations, further contributing to the overall burden of disease. Despite this, awareness of the disease in the general population and among healthcare professionals remains limited, likely due to a combination of an insufficient understanding of MASLD’s broader health impact and stigmatization [4]. Stigma has been recognized as a clinically meaningful psychosocial burden in several chronic conditions, particularly those perceived as preventable or lifestyle-related. Classic sociological work by Goffman conceptualizes stigma as a process by which individuals are discredited and reduced “from a whole and usual person to a tainted, discounted one,” while more recent models, such as Link and Phelan’s framework, emphasize stigma as a dynamic interplay between labeling, stereotyping, separation, status loss, and discrimination, embedded within broader social and structural power relations [5,6]. Stigmatization is not unique to MASLD but is a recurring theme in liver diseases more broadly. Liver diseases are often perceived as “low-prestige” conditions within both the healthcare system and society, making them particularly vulnerable to negative judgments regardless of etiology [7]. However, stigma in MASLD may be particularly pronounced due to its dual association with liver disease and metabolic risk factors such as obesity and type 2 diabetes, conditions that are themselves highly stigmatized [8,9]. Such dynamics not only contribute to delayed diagnosis, diminished quality of life, and inadequate treatment but may also result in insufficient communication and support from healthcare professionals [10]. Furthermore, the persistence of stigma is often reflected in limited access to accurate information, patient education, and disease-specific knowledge, leaving many patients without the resources needed to manage their condition effectively. Although emerging qualitative studies have explored the psychosocial consequences and symptom burden of MASLD [11,12,13,14], these studies have only partially addressed stigma. In particular, limited attention has been given to how different dimensions of stigma intersect in patients’ everyday lives and healthcare encounters.

This study, therefore, aims to explore how patients living with MASLD experience and interpret different dimensions of stigma across healthcare, social, and personal contexts. By addressing an important knowledge gap, the study contributes to a deeper understanding of the hidden psychosocial burden of MASLD and may inform strategies to reduce stigmatization and promote more patient-centered and equitable care.

### Theoretical Framework

The concept of stigma is widely used across disciplines and has been defined and conceptualized in multiple ways [5]. To provide a conceptual lens for understanding patients’ experiences, this study draws on the multidimensional framework proposed by Pryor and Reeder [6]. This framework conceptualizes stigma as operating at multiple interconnected levels: structural stigma (institutional and systemic conditions), public stigma (societal attitudes and stereotypes), self-stigma (internalization of negative beliefs), and stigma by association (experienced by those connected to a stigmatized individual). These levels interact and mutually reinforce processes of discrimination, exclusion, and status loss.

**Figure 1:**
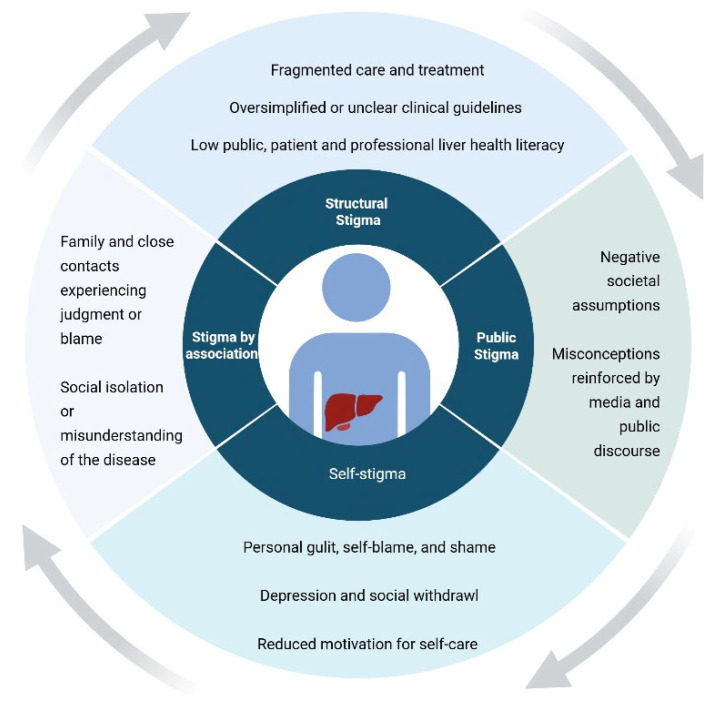
Conceptual model of stigma based on Pryor and Reeder’s framework [6].

Patients living with chronic conditions perceived as preventable or lifestyle-related, such as obesity, type 2 diabetes, substance use disorders, and mental illness, are particularly vulnerable to stigmatization. Within hepatology, liver diseases have historically been associated with moral judgment and low disease prestige [15,16]. While alcohol-related liver disease is often explicitly linked to moral evaluations of alcohol use, stigma in MASLD may be more ambiguous yet pervasive, rooted in assumptions about personal responsibility, overeating, physical inactivity, and lack of self-control. Such perceptions may affect psychological well-being, social relationships, and healthcare-seeking behavior, thereby reinforcing health inequities (Figure 1).

## 2. Materials and Methods

This study employed a qualitative design to investigate the lived experiences of stigma among individuals self-reporting a diagnosis of MASLD. The design was chosen to capture the nuanced perceptions, emotions, and coping strategies that may not be accessible through quantitative methods.

### 2.1. Data Collection

Data were collected using an anonymous online questionnaire administered in English and hosted on the secure web-based survey platform SurveyXact. The questionnaire consisted of 15 questions in total. Four questions addressed demographic characteristics, including gender, age group, country of residence, and time since diagnosis with MASLD. The remaining 11 questions were open-ended and explored participants’ experiences of stigmatization from family, healthcare professionals, and society; the perceived impact of these experiences; and the strategies participants used to respond to or manage stigma.

An anonymous online format was chosen to enable participation across geographical regions and to facilitate the disclosure of potentially sensitive experiences related to stigma. Given the emotionally vulnerable nature of the topic, anonymity was considered important to reduce social desirability bias and encourage open, reflective responses. While in-depth interviews may have allowed for further probing and elaboration of individual narratives, the online format provided accessibility and flexibility for participants from different countries.

The questionnaire was developed by the authors, drawing inspiration from prior qualitative studies of patient experiences with MASLD [11,12,13,14]. A shortened version of the questionnaire is presented in Table 1. The online format allowed participants to provide long-form responses in their own words, potentially enhancing disclosure of sensitive experiences [17]. Recruitment occurred via purposive sampling through the Canadian Liver Foundation’s Facebook page, where an open invitation disseminated the survey link. Eligibility criteria required participants to (1) self-report a diagnosis of MASLD, (2) be aged 18 years or older, and (3) be able to read and write in English. Diagnosis was based on self-report and was not independently clinically verified. No additional exclusion criteria were applied. Data collection took place between 19 November 2024 and 6 February 2025.

### 2.2. Data Analysis

Demographic variables were summarized using descriptive statistics. Categorical variables, including gender, age group, country of residence, and time since diagnosis with MASLD, were reported as percentages.

The study employed a descriptive qualitative design using an inductive thematic analysis approach as described by Braun and Clarke [18]. Coding and theme development were generated inductively from the questionnaire responses. The multidimensional stigma framework by Pryor and Reeder [6] was applied during the interpretative phase of the study to contextualize and discuss the results, not to structure initial coding or theme development.

All questionnaire responses were first read repeatedly to achieve familiarization with the data and gain an overall understanding of participants’ experiences. During this phase, initial notes were made regarding recurrent ideas, emotional expressions, and references to healthcare, stigma, and coping. Open coding was conducted at a semantic level, whereby each meaningful segment of the text (ranging from phrases to longer narrative responses) was assigned a descriptive code that closely reflected the participant’s own wording (e.g., “late or incidental diagnosis”, “fear of disease progression”, “blamed for lifestyle choices”, “dismissive healthcare communication”, and “peer support”). Coding was performed primarily using NVivo 14 software, with supplementary manual coding and code organization conducted in Microsoft Excel to allow for close comparison across responses. Codes were generated across the entire dataset and iteratively refined through constant comparison. Overlapping or redundant codes were merged, while broad or heterogeneous codes were subdivided into more specific subcodes to better capture nuance and variation within participants’ experiences. Throughout this process, codes were repeatedly reviewed against the original answers from the questionnaires to ensure they accurately represented participants’ meaning. Once coding was complete, related codes were clustered into preliminary themes. These candidate themes were reviewed and refined by examining their internal coherence and their distinctiveness from one another. This process resulted in the development of three overarching thematic domains that captured shared patterns across the dataset. Given the exploratory nature of the study and the use of an online questionnaire format, data collection was guided by pragmatic considerations rather than formal saturation criteria. However, during analysis, no substantially new themes emerged in the later responses, suggesting thematic sufficiency within the dataset. Themes and subthemes were then clearly defined and named. Representative quotations were selected to illustrate each theme. Quotations were chosen based on their clarity, richness, and ability to reflect experiences shared by multiple participants. All identifying information was removed to maintain participant confidentiality [18]. The research team consisted of healthcare professionals and researchers with experience in hepatology and qualitative health research. Their familiarity with MASLD provided contextual insight but may also have influenced sensitivity toward stigma-related narratives. To enhance reflexivity and analytical rigor, coding and theme development were conducted iteratively and discussed collaboratively. Interpretations were continuously checked against the original data to ensure that themes remained grounded in participants’ accounts.

### 2.3. Ethical Considerations

This study was performed according to the guidelines of the Helsinki Declaration. Due to the non-biomedical character of the study, no formal approval was required by the Danish National Center for Ethics (Sect. 14, subsection 2 of the Committees Act). Participants were assured of anonymity, confidentiality, and the voluntary nature of participation throughout the process. Informed consent was obtained electronically at the start of the questionnaire. All collected data were anonymized and handled in accordance with the EU’s General Data Protection Regulation (GDPR), 2016/679.

## 3. Results

A total of 23 participants completed the questionnaire. The sample was predominantly female and included participants from the United States, the United Kingdom, Canada, and Germany. Participants varied in age and time since diagnosis with MASLD. Detailed clinical and demographic characteristics are presented in Table 2.

### 3.1. Overview of the Analyses

A total of 24 pages of response text from the open-ended questionnaire were analyzed, resulting in 83 initial codes. Through iterative comparison and refinement, these codes were organized into three overarching thematic domains: (1) Diagnosis, Symptoms, and Medical Experiences; (2) Emotional and Social Impact; and (3) Coping Strategies and Advocacy. Themes were generated inductively from patterns identified across participants’ accounts. An example of the coding procedure from raw quotation to code, subtheme, and final theme is illustrated in Table 3.

### 3.2. Diagnosis, Symptoms, and Medical Experiences

Across participants, a dominant pattern concerned how MASLD was discovered and subsequently managed within healthcare settings. Many described receiving an incidental diagnosis during examinations for unrelated conditions, often without prior awareness of liver abnormalities, reinforcing the perception of MASLD as a “silent” condition. As one participant (female, USA, diagnosed <1 year ago) stated: “I was not aware I had it. I was diagnosed when having a CT scan for pneumonia. No symptoms that I am aware of.” Following diagnosis, many participants described receiving oversimplified or vague guidance from healthcare professionals, with “just lose weight” being the most frequently cited recommendation. Several participants noted that the information provided was not only inadequate but also inconsistent, with different healthcare professionals offering contradictory advice. One participant (female, UK, diagnosed 2 years ago) described: “A mishmash of misinformation from disagreeing physicians while trying to follow an extremely limited eating regime.” These narratives illustrated not only informational gaps but also conflicting medical advice, which contributed to confusion and frustration. Many participants further reported feeling blamed or implicitly judged, particularly in relation to weight, lifestyle, or presumed alcohol use. One participant (female, Canada, diagnosed 3 years ago) explained: “I’ve had to move provinces to obtain care.” Taken together, these accounts suggest that healthcare encounters were experienced not merely as medically limited but as emotionally impactful, shaping perceptions of legitimacy, support, and responsibility.

### 3.3. Emotional and Social Impact

Emotional distress emerged as a central and interwoven dimension of participants’ experiences. Across accounts, anxiety about disease progression, depressive symptoms, and diminished self-worth were frequently described. One participant (female, USA, diagnosed 1 year ago) reflected: “This diagnosis has plummeted any good feelings I have about myself.” Another (female, UK, diagnosed <1 year ago) shared: “At first, I felt so fat and unhealthy I wished I wasn’t alive.” Emotional responses were frequently described in relation to perceived judgment from others. Participants reported feeling blamed for their condition, particularly in relation to diet, body weight, and assumed alcohol consumption. Such experiences were described in interactions with healthcare professionals as well as with family members and friends. One participant (female, Germany, diagnosed 2 years ago) noted: “A previous physician assumed I was a drinker before I was properly diagnosed.” Similarly, another participant (male, USA, diagnosed 7 years ago) described how stigma extended beyond clinical settings: “People often don’t realize they don’t know the facts. Even healthcare professionals try to shame patients and sometimes succeed when I’m vulnerable.” Social interactions were often described as emotionally charged contexts. Several participants reported hiding their diagnosis to avoid misunderstanding or judgment. Others described strain in family or social situations centered around food and alcohol. For example, one participant (female, USA, diagnosed 7 years ago) explained: “Going out to eat with friends and they say I need to enjoy desserts and drinks. They don’t know what pain I’m in.” Another shared (female, USA, diagnosed 1 year ago): “Sometimes my family will comment on what and how much I am eating.” Across these narratives, stigma appeared to operate simultaneously at interpersonal and internalized levels, influencing both social participation and self-perception.

### 3.4. Coping Strategies and Advocacy

Despite challenges, participants described active efforts to manage both the medical and psychosocial dimensions of MASLD. A recurring pattern across responses was self-education and peer engagement as compensatory strategies for perceived gaps in professional support. One participant (female, Canada, diagnosed 3 years ago) stated: “We have a chat group and sometimes I think it helps people. You know you are not the only one with it.” Online communities were frequently described as providing validation, shared knowledge, and emotional reassurance. In contrast to experiences of dismissal in healthcare encounters, peer support was often characterized as empathetic and empowering. Participants’ experiences with lifestyle modifications varied. Some described successful dietary changes and improved clinical markers, whereas others emphasized the emotional, financial, or practical barriers to sustaining lifestyle adjustments. One participant (female, UK, diagnosed 1 year ago) reflected: “Changes are hard at my age.” Another explained: “Confusing and scary at the beginning. Having a support group helps.” Across accounts, coping was not limited to behavioral change but also involved advocacy and calls for more holistic care. Participants emphasized the need for integrated support addressing mental, emotional, and physical health. As one respondent summarized (female, USA, diagnosed 2 years ago), “A holistic approach to care, considering emotional, mental, and physical health, would promote a better quality of life.” These narratives suggest that patients position themselves not only as recipients of care but also as active agents navigating medical uncertainty, stigma, and self-management.

## 4. Discussion

This study illustrates how patients living with MASLD describe stigma as an integral dimension of their illness experience. Rather than occurring in isolated encounters, participants’ accounts suggest that stigma is embedded within clinical interactions, emotional responses, and everyday social contexts. Experiences of being blamed, dismissed, or provided with oversimplified guidance appeared to shape perceptions of legitimacy, support, and trust in healthcare relationships.

Consistent with earlier qualitative studies, participants described incidental diagnosis, conflicting medical advice, and limited communication as central challenges in MASLD care [11,12,13,14]. The frequently reported recommendation to “just lose weight” aligns with previous research in which lifestyle advice was perceived as insufficient and, at times, stigmatizing rather than supportive of sustainable change [3,12]. Similar dynamics have been observed in other chronic conditions perceived as lifestyle-related, including chronic obstructive pulmonary disease and obesity, where patients are frequently framed as personally responsible for their illness [19,20]. These parallels suggest that the experiences described in the present study may reflect broader patterns of moralization and responsibility embedded within healthcare practice.

Beyond clinical encounters, participants’ narratives point to a substantial emotional burden associated with MASLD. Reports of anxiety, shame, diminished self-worth, and social withdrawal are consistent with existing evidence demonstrating impaired health-related quality of life among patients with MASLD [4,21]. Importantly, the findings do not imply a direct causal pathway between stigma and psychological distress. Rather, they suggest that stigma-related experiences may interact with disease burden, prior vulnerabilities, and social context, potentially intensifying emotional strain. In this way, stigma appears woven into everyday illness management, shaping both how individuals interpret their condition and how they anticipate responses from others.

Participants’ descriptions of peer support and self-education highlight both resilience and structural gaps in formal care. Engagement in online communities appeared to provide validation and shared understanding, echoing research on digital peer support in chronic disease management [22]. At the same time, reliance on informal networks may reflect perceived deficiencies in continuity of care and access to tailored guidance. Calls for more holistic approaches integrating medical, emotional, and social dimensions resonate with emerging proposals for integrated care models in MASLD [11,23].

When viewed through Pryor and Reeder’s multidimensional stigma framework, participants’ accounts suggest the presence of interconnected forms of stigma. Public stigma was reflected in judgmental attitudes encountered in healthcare and social settings. Self-stigma emerged in descriptions of guilt, shame, and reduced self-worth. Structural stigma appeared in fragmented care pathways, limited disease-specific education, and simplified clinical narratives that positioned MASLD as primarily a consequence of personal lifestyle choices [7]. Although stigma by association was less explicitly articulated, strained family dynamics and social withdrawal indicate that relational dimensions may also be relevant.

Taken together, the findings suggest that stigma in MASLD operates across multiple levels that may reinforce one another. However, given the qualitative design, these interrelationships should not be interpreted as causal pathways. The study instead offers an interpretative understanding of how stigma is experienced and negotiated within broader illness trajectories and social environments. By foregrounding patients’ lived accounts, the findings contribute to a deeper appreciation of the hidden psychosocial dimensions of MASLD and underscore the importance of care models attentive to both biomedical and relational aspects of disease.

### 4.1. Relevance for Clinical Practice

Participants’ accounts of feeling blamed, dismissed, and insufficiently supported in healthcare encounters highlight stigma as a clinically relevant barrier to effective MASLD management. Experiences of oversimplified advice, conflicting information, and perceived moral judgment were described as undermining trust and discouraging engagement with care. These findings suggest that stigma is not merely an interpersonal concern but may influence adherence, help-seeking behavior, and emotional well-being.

**Figure 2:**
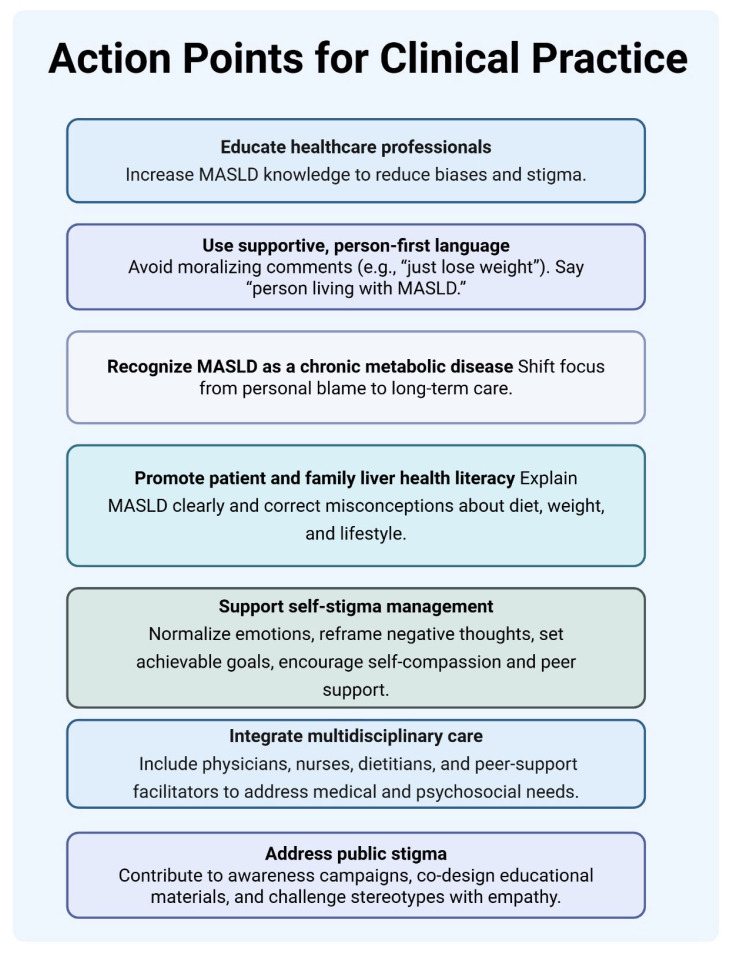
Approaches to reduce stigma in MASLD care.

Addressing stigma, therefore, requires attention at multiple levels, including everyday clinical communication and broader organizational practices [24]. Based on participants’ reported experiences, key approaches may include professional education to improve knowledge of MASLD [25,26], the use of non-stigmatizing and person-first language, promotion of liver health literacy [27,28], structured support for managing self-stigma, and multidisciplinary collaboration to integrate medical and psychosocial care [29]. Figure 2 summarizes these approaches.

Together, these recommendations emphasize that destigmatization begins within routine clinical interactions. By acknowledging patients’ experiences of blame and uncertainty and integrating stigma awareness into communication, education, and teamwork, healthcare professionals may contribute to more supportive and trust-enhancing care pathways. However, empirical evidence regarding the effectiveness of specific stigma-reduction strategies in MASLD remains limited, underscoring the need for further research.

### 4.2. Limitations

This study has several limitations that should be acknowledged. First, data were collected through an anonymous, online, open-ended questionnaire, which limited opportunities for the authors to clarify or probe responses. While this format allowed participants to reflect freely and disclose sensitive experiences, it may have constrained the depth and richness of the narratives compared with in-depth interviews or focus groups. In addition, as data collection was not formally guided by predefined saturation criteria, it cannot be determined whether additional answers or interviews would have yielded further nuances.

Second, the sample size was small, and recruitment relied on self-selection through a social media platform hosted by the Canadian Liver Foundation. As recruitment occurred via open online posts, it was not possible to determine how many individuals were exposed to the invitation, and a response rate could therefore not be calculated. This recruitment strategy may have introduced selection bias, as participants were likely to be individuals who were socially engaged online, motivated to share their experiences, and potentially more psychologically affected by stigma. As a result, the findings may overrepresent patients with strong emotional responses or prior negative healthcare experiences and may not fully capture the perspectives of patients who are less engaged, less distressed, or less inclined to participate in online research.

Third, as this study employed a qualitative design, the findings are intended to provide in-depth insights into participants’ lived experiences rather than statistical generalization. The results should therefore be interpreted as contextually grounded and exploratory. Fourth, although basic demographic information such as age, gender, and country of residence was collected, more detailed sociodemographic and clinical characteristics (e.g., body mass index, disease stage, or socioeconomic status) were not systematically assessed and are therefore not reported. This limits the ability to examine how experiences of stigma may vary across clinical subgroups or social positions. Moreover, participants were recruited from different countries, including Canada, Germany, the United Kingdom, and the United States. Cultural norms, public health discourses, and differences in healthcare systems may shape how stigma is experienced and negotiated. As a result, the findings may reflect context-specific dynamics and should be interpreted with caution when considering transferability to other settings. Furthermore, all participants self-reported a diagnosis of MASLD, which could not be independently clinically verified. This reliance on self-report may introduce misclassification, although recruitment through a liver disease advocacy organization increases the likelihood that participants had received a formal diagnosis.

Finally, as in all qualitative research, the analysis is shaped by the authors’ interpretations. However, steps such as iterative coding, the use of an established analytic framework, and discussions among the authors were employed to enhance credibility and analytic rigor. Despite these limitations, the study provides important exploratory insights into how stigma is experienced and negotiated by patients living with MASLD and highlights areas for improvement in clinical practice that warrant further investigation using larger and more diverse samples.

### 4.3. Future Research

Future research should examine stigma in MASLD using larger and more diverse samples, including patients across clinical disease stages and sociodemographic groups. Combining qualitative and quantitative approaches may help clarify how stigma relates to mental health outcomes, healthcare engagement, and disease management over time. In addition, intervention studies are needed to evaluate the effectiveness of stigma-reduction strategies in MASLD care, including training for healthcare professionals, multidisciplinary care models, and patient education initiatives. Previous work indicates that interventions targeting stigma are emerging but currently limited in scope and generalizability, and that feasibility and acceptability outcomes are promising yet require testing in larger efficacy trials [30,31]. Finally, comparative studies across different etiologies of liver disease may help identify both shared and context-specific stigma mechanisms and inform targeted, context-sensitive approaches.

## 5. Conclusions

This study illustrates the complex and often distressing reality of living with MASLD and how stigma is described as shaping the lived experiences of patients, not only through healthcare interactions, public stereotypes, and structural barriers but also via self-stigma. Participants consistently reported feeling blamed, dismissed, or receiving oversimplified and vague guidance from healthcare professionals, which undermined trust and reinforced a sense of being unsupported. Stigma further affected emotional well-being, with many describing depression, shame, and social isolation. While some sought support through peer groups and self-education, lifestyle changes remained difficult without accessible professional guidance and comprehensive support systems. Overall, the findings highlight a clear need for more holistic care approaches to reduce stigmatization. Addressing all dimensions of stigma will be essential to improving equity of care for patients living with MASLD and to alleviating what may be described as the hidden burden of MASLD.

## Figures and Tables

**Table 1:** Open-ended questions (shortened for manuscript presentation).

Thematic Focus	Open-Ended Questions
Lived experience of MASLD	Can you describe your experience of living with liver disease?
Emotional and daily life impact	How have these experiences affected your emotional well-being or daily life?
Experience of judgment and blame	Have you ever felt judged or blamed for having fatty liver disease? Can you share an example
Social relationships	Have friends or family treated you differently because of your diagnosis? Can you share an example
Public settings	Can you describe a time when you felt judged in a public setting (e.g., workplace or social event)?
Healthcare communication	How have healthcare professionals communicated with you about your condition?
Perceived blame from healthcare professionals	Have you ever felt blamed or judged by a healthcare professional?
Self-perception and shame	Since your diagnosis, how has your self-perception changed? Do you ever feel guilty or ashamed about your condition?
Coping strategies	What strategies have helped you cope with any negative feelings or judgments related to your condition?
Support needs	What kind of support would reduce the burden of stigmatization for patients like you?
Additional reflections	Is there anything else you would like to share?

**Table 2:** Participants’ clinical and demographic characteristics.

Gender, N (%)	
Female	21 (91)
Men	2 (9)
Age, N (%)	
35–50 years	10 (43)
51–60 years	10 (43)
61–70+ years	3 (14)
Country of residence, N (%)	
United States	11 (48)
United Kingdom	5 (22)
Canada	5 (22)
Germany	2 (8)
Time since diagnosis of MASLD, N (%)	
Less than one year	6 (26)
One year	7 (30)
Two years	3 (13)
Three years	3 (13)
Seven years	2 (9)
More than ten years	2 (9)

**Table 3:** Example of the coding process.

Raw Quotation	Initial Code	Subtheme	Theme
“I was not aware I had it. Was diagnosed when having a CT scan for pneumonia. No symptoms that I am aware of.”	Incidental diagnosis	Late diagnosis and lack of awareness	Diagnosis, Symptoms, and Medical Experiences
“A mishmash of misinformation from disagreeing doctors while trying to follow an extremely limited eating regime.”	Conflicting medical advice	Medical misinformation and frustration	Diagnosis, Symptoms, and Medical Experiences
“This diagnosis has plummeted any good feelings I have about myself.”	Reduced self-worth	Mental health struggles	Emotional and Social Impact
“We have a chat group and sometimes I think it helps people. You know you are not the only one with it.”	Peer support	Self-advocacy and education	Coping Strategies and Advocacy

## Data Availability

The data that support the findings of this study are available from the corresponding author upon reasonable request to protect participant confidentiality.

## Abbreviations

The following abbreviations are used in this manuscript:

MASLD	Metabolic dysfunction-associated steatotic liver disease
NAFLD	Non-alcoholic fatty liver disease
GDPR	General Data Protection Regulation

## References

[1] Chan W.K., Chuah K.H., Rajaram R.B., Lim L.-L., Ratnasingam J., Vethakkan S.R. (2023). Metabolic dysfunction-associated steatotic liver disease (MASLD): A state-of-the-art review. J. Obes. Metab. Syndr..

[2] Teng M.L., Ng C.H., Huang D.Q., Chan K.E., Tan D.J., Lim W.H., Yang J.D., Tan E., Muthiah M.D. (2023). Global incidence and prevalence of nonalcoholic fatty liver disease. Clin. Mol. Hepatol..

[3] Younossi Z., Tacke F., Arrese M., Sharma B.C., Mostafa I., Bugianesi E., Wong V.W.-S., Yilmaz Y., George J., Fan J. (2019). Global perspectives on nonalcoholic fatty liver disease and nonalcoholic steatohepatitis. Hepatology.

[4] Younossi Z.M., Kalligeros M., Henry L. (2025). Epidemiology of metabolic dysfunction-associated steatitic liver disease. Clin. Mol. Hepatol..

[5] Goffman E. (1963). Stigma: Notes on the Management of Spoiled Identity.

[6] Bos A.E.R., Pryor J.B., Reeder G.D., Stutterheim S.E. (2013). Stigma: Advances in theory and research. Basic Appl. Soc. Psychol..

[7] Album D., Westin S. (2008). Do diseases have a prestige hierarchy? A survey among physicians and medical students. Soc. Sci. Med..

[8] Ramírez-Mejia M.M., Qi X., Abenavoli L., Mendez-Sanchez N. (2024). The myth of the stigma of fatty liver: What does the evidence show?. Ann. Hepatol..

[9] Sharrock K., Cross T.J.S., Hebditch V., Hollywood C. (2025). Impact of stigma on individuals living with liver diseases and why it matters. Frontline Gastroenterol..

[10] Westbury S., Oyebode O., van Rens T., Barber T.M. (2023). Obesity stigma: Causes, consequences, and potential solutions. Curr. Obes. Rep..

[11] Swain M.G., Pettersson B., Meyers O., Venerus M., Oscarsson J. (2023). A qualitative patient interview study to understand the experience of patients with nonalcoholic steatohepatitis. Hepatol. Commun..

[12] Shinde S., Taylor N., Chinthammit C., Wilson R., Burgess S.M., Poon J.-L. (2024). Understanding the impact of non-alcoholic steatohepatitis with metabolic comorbidities on adults: A real-world qualitative study. Curr. Med. Res. Opin..

[13] Cook N.S., Nagar S.H., Jain A., Balp M.-M., Mayländer M., Weiss O., Chatterjee S. (2019). Understanding patient preferences and unmet needs in non-alcoholic steatohepatitis (NASH): Insights from a qualitative online bulletin board study. Adv. Ther..

[14] Avery L., Exley C., McPherson S., Trenell M.I., Anstee Q.M., Hallsworth K. (2017). Lifestyle behavior change in patients with nonalcoholic fatty liver disease: A qualitative study of clinical practice. Clin. Gastroenterol. Hepatol..

[15] Kane J.C., Elafros M.A., Murray S.M., Mitchell E.M.H., Augustinavicius J.L., Causevic S., Baral S.D. (2019). A scoping review of health-related stigma outcomes for high-burden diseases in low- and middle-income countries. BMC Med..

[16] Corrigan P.W., Watson A.C. (2002). Understanding the impact of stigma on people with mental illness. World Psychiatry.

[17] Stebbins R.A. (2001). Exploratory Research in the Social Sciences.

[18] Braun V., Clarke V. (2006). Using thematic analysis in psychology. Qual. Res. Psychol..

[19] Sohn J., Rochester E., Oluyase A.O. (2025). Features of COPD that lead to stigmatization and its consequences: A framework synthesis. COPD.

[20] Crompvoets P., Nieboer A.P., Rossum E.F.C., Cramm J.M. (2024). Perceived weight stigma in healthcare settings among adults living with obesity: A cross-sectional investigation of the relationship with patient characteristics and person-centered care. Health Expect..

[21] Kennedy-Martin T., Bae J.P., Paczkowski R., Freeman E. (2018). Health-related quality of life burden of nonalcoholic steatohepatitis: A robust pragmatic literature review. J. Patient Rep. Outcomes.

[22] Yeo G.H., Loo G., Oon M., Pang R., Ho D. (2023). A digital peer support platform to translate online peer support for emerging adult mental well-being: Randomized controlled trial. JMIR Ment. Health.

[23] Chalasani N., Younossi Z., LaVine J.E., Charlton M., Cusi K., Rinella M., Harrison S.A., Brunt E.M., Sanyal A.J. (2018). The diagnosis and management of nonalcoholic fatty liver disease: Practice guidance from the American Association for the Study of Liver Diseases. Hepatology.

[24] European Association for the Study of the Liver, American Association for the Study of Liver Disease, Latin American Association for the Study of the Liver, Asian Pacific Association for the Study of the Liver (2023). Ending stigmatizing language in alcohol and liver disease: A liver societies’ statement. J. Hepatol..

[25] Talumaa B., Brown A., Batterham R., Kalea A.Z. (2022). Effective strategies in ending weight stima in healthcare. Obes. Rev..

[26] Vaz J., Willemse J., Jepsen P. (2024). Addressing the impact of stigma in liver diseases: A call for proper language and responsibility allocation. J. Hepatol..

[27] Corrigan P.W., Rao D. (2012). On the self-stigma of mental illness: Stages, disclosure, and strategies for change. Can. J. Psychiatry.

[28] Shahwan S., Goh C.M.J., Tan G.T.H., Ong W.J., Chong S.A., Subramaniam M. (2022). Strategies to reduce mental illness stigma: Perspectives of people with lived experience and caregivers. Int. J. Environ. Res. Public Health.

[29] European Association for the Study of the Liver (EASL), European Association for the Study of Diabetes (EASD), European Association for the Study of Obesity (EASO) (2024). EASL-EASD-EASO Clinical Practice Guidelines on the management of metabolic dysfunction-associated steatotic liver disease (MASLD). J. Hepatol..

[30] Akyirem S., Ekpor E., Batten J., Brady V. (2024). Reducing health-related stigma in adults living with chronic non-communicable disease: A systematic review and meta-analysis. Soc. Sci. Med..

[31] Pearl R.L., Saunders D., Groshon L.C., Li Y., Shonrock A., Puhl R.M., Driscoll K.A., Manavalan P., Gelfand J.M., Wadden T.A. (2025). Feasibility and acceptability of a psychological intervention for internalized health-related stigma among adults with chronic health conditions: Preliminary investigation. JMIR Form. Res..

